# 

**Published:** 1815-03

**Authors:** 


### MONTHLY CATALOGUE OF MEDICAL BOOKS.

OBSERVATIONS on the Symptoms and Treatment of the Diseased Spine, more particularly relating to the incipient Stages; with some Remarks on the consequent Palsy. By Thomas Copeland, Esq. Fellow of the Royal College of Surgeons, and Assistant-Surgeon to the Westminster General Dispensary. 8vo. —Callow.

The Medical Friend to the Asthmatic Patient, shewing him the particular Species of his Disorder, and also what to pursue, and what to avoid, in regard to Medicine, Diet, Situation, and other circumstances connected with his Complaint. By W. Nisbet, M.D. 12mo.—Callow.

Observations on a Case of Strabismus Divergens, or Squinting. By M. Roux. 12mo.—Cox.

Engravings of the Thoracic and Abdominal Viscera, and the Canals connected with them; representing the Natural Appearance of those important Parts immediately after Death, and without being affected by previous Disease. By Alexander Monro, jun. 4to.—Longman and Co.

A. Corn. Celsus of Medicine in Eight Books, translated, with Notes, critical and explanatory. By James Greive, M.D. A new edition. 12mo.—Anderson.

An Attempt to establish a pure Scientific System of Mineralogy. By J. J. Berzelius, M.D. Translated from the Swedish, by John Black. 8vo.—Baldwin and Co.

---

Want of room obliges us to defer the MONTHLY PRICES OF DRUGS; but, by reference to those given in our January Number, and by the aid of the following subsequent variations, our readers will ascertain their correct value, as at Messrs. SELWAY and HENLEY's Chemical and Pharmaceutical Laboratory, 35, Upper Mary-le-bone Street, Portland Place.

| | | | | | |
|---|---|---|---|---|---|
| A[illegible]go ........ lb. | 7 | 0 | Lichen Islandicus ...... P. lb. | 1 | 6 |
| Argenti Nitras ........ | 6 | 9 | Manna optima ........ | 7 | 0 |
| Assafœtida Gummi-resina .. | 5 | 6 | Moschus pod, 24s. in gr. unc. | 42 | 0 |
| Balsamum Peruvianum ...... | 28 | 0 | Opium (Turkey) ........ | 29 | 0 |
| ——— Tolutanum ...... | 18 | 0 | ——— (East India) ...... lb. | 36 | 0 |
| Coccinella ........ | 52 | 0 | Potassa Subcarbon ........ | 1 | 8 |
| Colocynthidis ........ | 7 | 3 | Rosæ Folia ........ | 7 | 6 |
| Croci stigmata ........ | 62 | 0 | Rhæi Radix (East India) .... | 16 | 0 |
| Galbani Gummi-resinæ .... | 8 | 0 | Sarsaparilla Radix Inc........ | 5 | 6 |
| Hydrargyrus purificatus .... | 5 | 10 | Spiritus Vini rectificatus .... | 25 | 0 |
| Liquor Ammoniæ .... P.L. | 3 | 0 | Sulphur ........ | 0 | 8 |
| ——— Vol. Corn. Cerv. .... | 1 | 2 | Tinctura Cast. ........ | 8 | 6 |

---

### TO CORRESPONDENTS.

*We have received a Communication from our former correspondent Dr. Walker of Huddersfield; but, as the superscription is different from the place where it was received, we wait to know whether there is an error, that we may either insert, or forward it to another publication.*

*Communications from Drs. Kinglake and Robertson; Messrs. F. E. Franklyn, Wilson, C. W. Smerdon, J. Lignum, &c. &c. have also come to hand.*

